# Feasibility and Acceptability of an mHealth ACP Tool in Primary Care

**DOI:** 10.1093/geroni/igab046.1991

**Published:** 2021-12-17

**Authors:** Desh Mohan, Katelin Cherry, Tatiana Fofanova, Taylor Huffman, Glenn Davis, Anthony Comito, Elissa Kozlov

**Affiliations:** 1 Koda Health, Houston, Texas, United States; 2 Cypress Physicians Association, Spring, Texas, United States; 3 FOUNDRY41, Houston, Texas, United States; 4 Rutgers University, Piscataway, New Jersey, United States

## Abstract

With only 7% of Medicare beneficiaries having completed Advance Care Planning with their physicians, engagement in Advance Care Planning in the clinical setting has been historically low. This study investigated the feasibility of introducing the Koda Health Advance Care Planning software platform in the primary care setting, and whether patients would engage in advance care planning through this medium. The Koda platform is a video-driven, web application that guides patients through advance care planning concepts, including values and quality of life exploration, surrogate decision maker selection, life-support treatments, and advance directive completion. The study was completed over a six-month period in two primary care clinics in the Houston, Texas area. Inclusion criteria were age 55 or older, English-speaking, and capacity for medical decision making. 339 patients met eligibility criteria and had a median age of 73 (range 59-89). All participants were offered the platform, and 262 (77%) created an account and began planning for their care. Of the patients that created an account, 87% completed all ACP steps on the platform and 72% identified a surrogate decision maker. The median time spent on the platform was 18 minutes. The Koda platform appears to be a useful tool for patients and providers to improve engagement in advance care planning and improve surrogate decision maker identification. Further research is needed to understand whether the Koda platform aids in providing goal-concordant care.

